# Effects of a six-week combined tire-squat and explosive-kicking programme on roundhouse-kick performance in highly trained male Muay Thai athletes: a parallel-group controlled trial

**DOI:** 10.3389/fspor.2026.1885080

**Published:** 2026-08-25

**Authors:** Raja Syed Tengku Sulaiman

**Affiliations:** Major in Physical Education, School of Education, Walailak University, Nakhon Si Thammarat, Thailand

**Keywords:** combat sports, Muay Thai, neuromuscular adaptations, repeated-kicking capacity, roundhouse-kick performance, tire-squat training

## Abstract

**Background:**

Muay Thai is a high-intensity striking sport requiring repeated roundhouse kicks performed with speed, force, and precision under fatigue. Although lower-limb resistance training is widely used in combat-sport conditioning, evidence for combined sport-specific programmes remains limited. This study investigated the effects of a six-week combined tire-squat and explosive-kicking programme on roundhouse-kick performance in highly trained male Muay Thai athletes.

**Methods:**

Thirty-nine highly trained male Muay Thai athletes (15–25 years) participated in this parallel-group controlled trial. Participants were allocated to an experimental group (EXP, *n* = 20), which completed a six-week combined tire-squat and explosive-kicking programme in addition to usual training, or a control group (CON, *n* = 19), which continued usual training. Outcomes included stimulus-to-contact time, kick accuracy, repeated-kicking performance decrement (fatigue index; an indicator of repeated-kicking capacity), kicking consistency, dynamic balance, and inter-limb asymmetry. Longitudinal changes were analysed using linear mixed-effects models.

**Results:**

Significant group × time interactions were observed for stimulus-to-contact time, repeated-kicking performance decrement, and kicking consistency (all BH-adjusted *p* = 0.001). Compared with CON, EXP showed greater improvements in stimulus-to-contact time (−0.05 s; *g* = 1.45), repeated-kicking performance decrement (−3.2%; *g* = 0.95), and kicking consistency (−1.8 percentage points; *g* = 0.88). A small interaction was observed for kick accuracy (unadjusted *p* = 0.048; BH-adjusted *p* = 0.072; *g* = 0.28); however, it did not remain statistically significant after Benjamini–Hochberg adjustment. No significant interactions were observed for dynamic balance (BH-adjusted *p* = 0.221) or inter-limb asymmetry (BH-adjusted *p* = 0.142).

**Conclusions:**

A six-week combined tire-squat and explosive-kicking programme was associated with improvements in stimulus-to-contact time, repeated-kicking capacity (reflected by a lower repeated-kicking performance decrement index), and kicking consistency in highly trained Muay Thai athletes. The programme was associated with improvements in selected aspects of repeated roundhouse-kick performance. Because the intervention combined tire-squat exercise with explosive-kicking practice, the independent effects of each component could not be determined.

## 1 Introduction

Muay Thai is an intermittent, high-intensity striking sport that requires athletes to repeatedly execute rapid, explosive, and well-coordinated kicking actions under considerable physiological and mechanical stress (1, 2). Among these techniques, the roundhouse kick is regarded as one of the most decisive because it relies on effective lower-limb force production, proximal-to-distal segmental coordination, postural control, and efficient transfer of momentum towards the target (3). Biomechanical studies have shown that effective roundhouse kicking is characterised by rapid execution, coordinated pelvic and hip movement, and efficient target contact, all of which contribute to successful performance in high-level striking sports (3). Accordingly, roundhouse-kick performance may be understood as a multidimensional construct encompassing technical proficiency, repeated-effort capacity, movement control, and the ability to maintain performance during repeated high-intensity efforts (1–3).

Resistance training is widely used to enhance sport-specific performance, particularly when the training stimulus reflects the mechanical and temporal characteristics of the target movement (4, 5). A recent systematic review and meta-analysis reported substantial benefits of resistance training for sport-specific performance in highly trained athletes, supporting its role as an effective supplementary training modality (4). These findings are particularly relevant to combat sports, where the ability to generate force rapidly and repeatedly may influence the quality of striking actions throughout a bout (1, 2, 4). Emerging evidence further suggests that resistance-training adaptations may be especially relevant in highly trained athletes, for whom relatively small improvements in force production, coordination, and rate of force development may influence sport-specific performance (5, 6).

High-intensity interval training (HIIT) is also highly relevant to combat sports because these disciplines involve repeated bouts of high-intensity effort interspersed with brief recovery periods (1, 2). Systematic reviews and meta-analyses have demonstrated that HIIT improves both aerobic- and anaerobic-related performance outcomes in combat-sport athletes (1, 2). Within this context, interval-based training structures appear well aligned with the physiological demands of striking sports and may therefore provide an appropriate framework for developing repeated high-intensity kicking performance.

From an applied perspective, combining progressive lower-limb resistance exercise with explosive sport-specific kicking practice may provide a practical training approach that integrates these complementary training principles. Tire-squat exercise provides a lower-limb resistance stimulus while emphasising trunk stability, hip and knee extension, and repeated force production under functional loading conditions (6). When combined with repeated explosive roundhouse-kick practice within an interval-based training structure, this approach may facilitate the transfer of lower-body physical qualities to sport-specific kicking performance by integrating progressive resistance exercise with repeated technical execution under incomplete recovery (1, 6). Internal training load can also be monitored pragmatically using session rating of perceived exertion (session RPE), which is widely applied in high-performance sport settings.

Despite these practical and theoretical considerations, evidence regarding combined lower-limb resistance and sport-specific kicking interventions designed to improve roundhouse-kick performance in highly trained Muay Thai athletes remains limited. Previous research has shown that acute post-activation potentiation induced by heavy squatting can enhance roundhouse-kick power, suggesting that lower-body force-oriented exercise may influence selected kicking-related outcomes in trained fighters (7). In addition, recent work using the Frequency Speed Kick Test (FSKT) has highlighted the importance of repeated-kicking performance during intermittent striking activity (8). Findings from the broader combat-sport literature further indicate that movement execution speed, target-directed control, and repeated-kicking performance represent important dimensions of striking performance (3, 7, 8). However, intervention research in combat sports continues to be challenged by heterogeneous training protocols, inconsistent outcome definitions, and practical constraints that may limit causal attribution and cross-study comparability. These challenges highlight the need for standardized, reproducible, and context-sensitive experimental approaches capable of generating more robust and translatable evidence for sport-specific practice (9).

A further consideration relates to outcome selection. In applied Muay Thai settings, reliance on laboratory-based impact-force assessment may not always be practical because of limited access to specialised instrumentation. A multidimensional field-based assessment approach may therefore provide a more ecologically relevant evaluation of sport-specific performance under applied training conditions. Accordingly, the present study assessed stimulus-to-contact time, kick accuracy, repeated-kicking capacity (operationalized as a repeated-kicking performance decrement index), kicking consistency, dynamic balance, and inter-limb asymmetry, thereby capturing complementary dimensions of roundhouse-kick performance (3, 8, 10–12). Dynamic balance is particularly relevant because unilateral kicking requires stable control of the support limb, whereas inter-limb asymmetry may provide additional information regarding movement efficiency and lower-limb coordination (10–12).

Accordingly, the present study investigated the effects of a six-week combined tire-squat and explosive-kicking programme on selected measures of roundhouse-kick performance in highly trained male Muay Thai athletes using a parallel-group controlled design. It was hypothesised that participants allocated to the combined training programme would demonstrate greater improvements in stimulus-to-contact time, repeated-kicking capacity, and kicking consistency than participants who continued their usual Muay Thai training alone.

## 2 Materials and methods

### 2.1 Study design

This study employed a parallel-group controlled trial with pre- and post-intervention assessments. Participants were allocated to either an experimental group (EXP), which completed a six-week combined tire-squat and explosive-kicking programme in addition to their usual Muay Thai training, or a control group (CON), which continued their usual Muay Thai training alone (4, 13).

Following eligibility screening, 39 participants were allocated using a manual lottery-based allocation procedure conducted by the principal investigator with assistance from a trained research assistant before the start of the intervention. Each eligible participant was assigned a unique identification number, which was written on an individual table-tennis ball and placed into an opaque container. After thorough mixing, the balls were drawn sequentially, and participants were assigned alternately to Group 1 and Group 2 according to the order in which their identification numbers were drawn, resulting in 20 participants in the experimental group (EXP) and 19 participants in the control group (CON). Following completion of the allocation procedure, Group 1 was designated as the experimental group and Group 2 as the control group. Participants were informed of their assigned study group immediately after allocation. Because no formal allocation concealment mechanism was implemented, the absence of allocation concealment is acknowledged as a methodological limitation. Furthermore, because the manual lottery-based allocation procedure was performed without allocation concealment, the possibility of selection bias cannot be completely excluded. Nevertheless, predefined eligibility criteria, standardized participant screening, and a prespecified allocation procedure were applied consistently to all participants to minimise this potential source of bias.

No participants were lost to follow-up after the intervention commenced, and all allocated participants completed the study and were included in the final statistical analyses.

Owing to the nature of the exercise intervention and the visible differences between the training protocols, participants and training personnel could not be blinded to group allocation. Outcome assessments were performed by the same assessor using standardized procedures at both PRE and POST testing sessions to ensure consistency across measurement occasions. However, complete assessor blinding was not feasible in this field-based setting. Accordingly, the possibility of measurement bias associated with non-blinded outcome assessment is acknowledged as a methodological limitation.

The intervention was conducted over six weeks, with outcome assessments performed 48–72 h before (PRE) and 48–72 h after (POST) completion of the intervention. All testing sessions were conducted at approximately the same time of day (±1 h) under standardized environmental conditions to minimise potential circadian and contextual influences on performance. Participants were instructed to maintain their usual Muay Thai training outside the intervention, avoid strenuous exercise for 24–48 h before each testing session, maintain consistent dietary and hydration practices, and attend both testing sessions under comparable conditions.

The primary analytical focus was the group × time interaction, representing the effect of the intervention relative to usual training. This interaction was examined using linear mixed-effects models, with participant included as a random intercept to account for within-subject dependence (14). Model assumptions were evaluated by visual inspection of residual distributions and homoscedasticity, and outcome variables were transformed where necessary to improve model fit. Pairwise comparisons were adjusted using the Benjamini–Hochberg false discovery rate (FDR) procedure where appropriate to account for multiple comparisons (15). Statistical significance was set at *p* < 0.05 (two-tailed). Statistical analyses were performed by the principal investigator using standardized analytical procedures. Although the analyses followed a prespecified statistical plan, the absence of an independent blinded statistician is acknowledged as a potential source of analytical bias and is discussed in the Limitations section.

The study was designed, conducted, and reported in accordance with the CONSORT 2025 Statement and its accompanying Explanation and Elaboration guidance for randomized and controlled intervention studies, where applicable (16, 17).

### 2.2 Participants

Forty-four highly trained male Muay Thai athletes aged 15–25 years were initially screened for eligibility from professional training camps in Southern Thailand. Before allocation, five athletes were excluded from participation: one because of a recent musculoskeletal injury, three because they had been selected to attend a professional training camp in preparation for ONE Championship competition, and one because of personal reasons. The remaining 39 eligible athletes were allocated using the lottery-based allocation procedure described in Sec. 2.1, resulting in 20 participants in the experimental group (EXP) and 19 participants in the control group (CON). No participants were lost to follow-up after the intervention commenced, and all allocated participants completed the study and were included in the final statistical analyses.

Eligibility criteria included at least three years of structured Muay Thai training, regular participation in a minimum of four training sessions per week, and no lower-limb injury during the preceding three months. Athletes were excluded if they had a current musculoskeletal injury, known neurological or vestibular disorders, or were undertaking additional lower-limb strength or power training outside the study protocol. An additional exclusion criterion was absence from more than 15% of the prescribed intervention sessions. No participant met this criterion during the intervention period. Consequently, all allocated participants completed the six-week intervention and were included in the final analyses. Intervention adherence is reported in the Results section, with additional details provided in the Supplementary Material.

All participants provided written informed consent before enrolment. For participants younger than 18 years, written parental consent together with participant assent was obtained before study participation. The study was conducted in accordance with the principles of the Declaration of Helsinki and was approved by the Human Research Ethics Committee of Walailak University (Approval No. WUEC-25-158-01).

**Figure 1:**
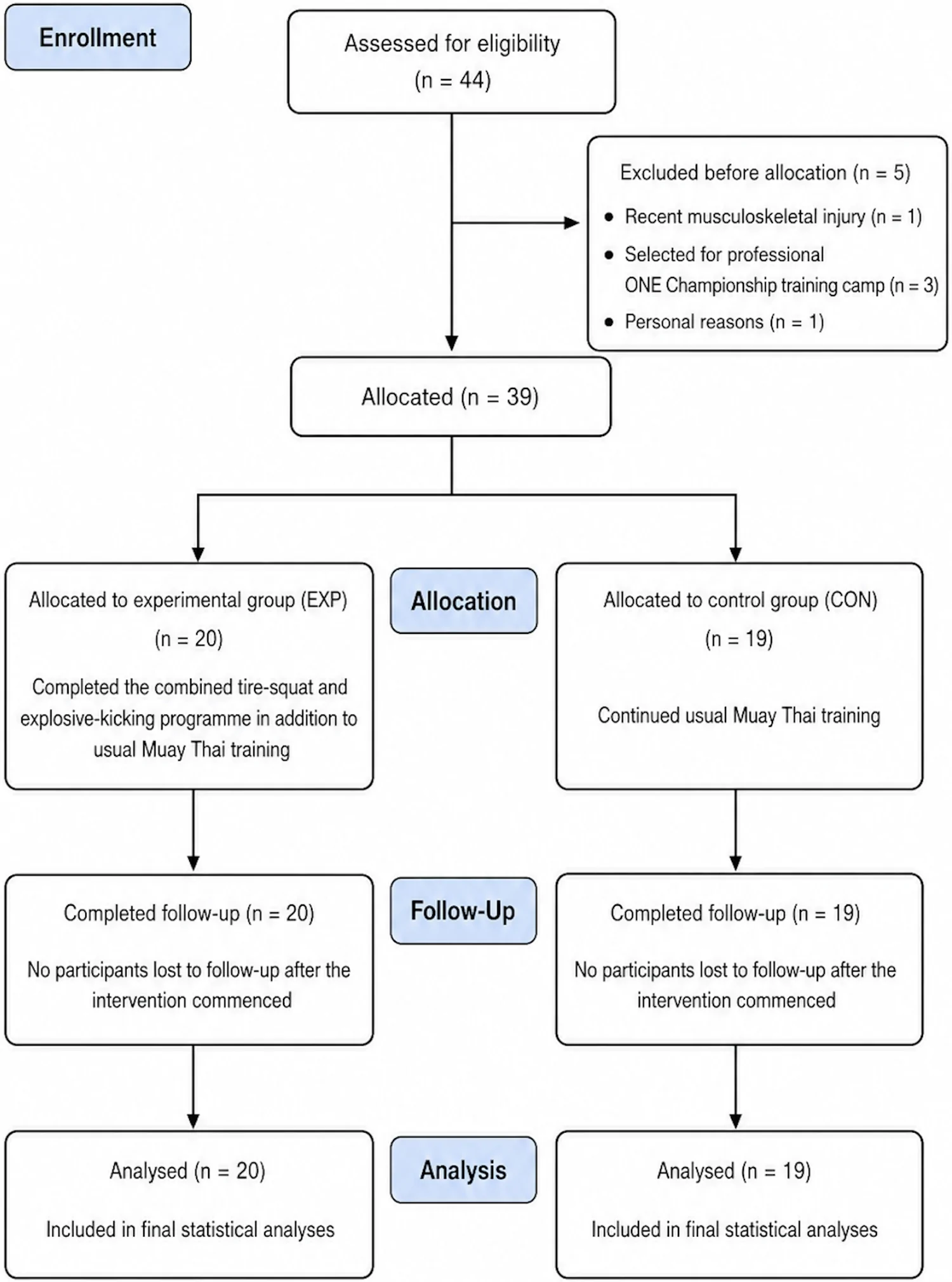
CONSORT 2025 flow diagram illustrating participant screening, exclusions, allocation, follow-up, and final analyses. A total of 44 highly trained male Muay Thai athletes were assessed for eligibility. Five athletes were excluded before allocation: one because of a recent musculoskeletal injury, three because of selection for a professional ONE Championship training camp, and one for personal reasons. The remaining 39 eligible participants were allocated to the experimental group (EXP, *n* = 20) or the control group (CON, *n* = 19). No participants were lost to follow-up after the intervention commenced, and all allocated participants completed the study and were included in the final statistical analyses.

Participant progression through screening, allocation, follow-up, and analysis is presented in the CONSORT flow diagram (Figure 1).

### 2.3 Procedures

Participants completed a familiarization session at least 48 h before baseline testing. During both PRE and POST testing sessions, outcome assessments were performed in a standardized order to minimise potential fatigue and order effects: dynamic balance, stimulus-to-contact time, kick accuracy, and repeated-kicking performance decrement (fatigue index), used as an indicator of repeated-kicking capacity (18–20). All measurements were performed by the same assessor under standardized environmental conditions.

The experimental group (EXP) completed a six-week high-intensity tire-squat training programme comprising three sessions per week on non-consecutive days, whereas the control group (CON) continued their usual Muay Thai training alone. Each training session in the EXP group consisted of a standardized warm-up (10–12 min), high-intensity tire-squat training, and, from week 3 onwards, integrated explosive roundhouse-kick drills (18, 19).

A large tractor tire served as the external resistance and was held in a bear-hug position throughout the squat exercise. Participants performed squats to approximately 90° of knee flexion while maintaining trunk stability and appropriate lower-limb alignment throughout each repetition. The training programme progressed from four sets of eight repetitions at moderate intensity (RPE 5–6) during weeks 1–2, to five sets of six repetitions at moderate-to-high intensity (RPE 6–7) during weeks 3–4, and finally to six sets of five repetitions at high intensity (RPE 7–8) during weeks 5–6 (19, 20). Explosive alternating roundhouse kicks were performed 20–30 s after each tire-squat set, with inter-set recovery periods of 90–150 s.

External load was increased by approximately 5%–10% when participants completed all prescribed repetitions with correct technique and reported a session rating of perceived exertion (session RPE) of ≤7. Conversely, the load was reduced if movement quality deteriorated or perceived exertion became excessive (19). Training tolerance was monitored using session RPE (CR-10 scale), attendance across the 18 planned intervention sessions, perceived fatigue, muscle soreness, and the occurrence of adverse events. No major protocol deviations occurred during the intervention period, and the intervention was delivered as planned without modification. No serious intervention-related adverse events were observed. Mild, transient muscle soreness was occasionally reported during the early weeks of training but resolved spontaneously without medical treatment or interruption of the intervention.

### 2.4 Outcome measures

The study evaluated six Muay Thai-specific performance outcomes: stimulus-to-contact time, kick accuracy, repeated-kicking performance decrement (fatigue index), kicking consistency, dynamic balance, and inter-limb asymmetry (21–23). Outcome measures were specified before data analysis according to the study protocol. Stimulus-to-contact time was designated as the principal performance outcome, whereas repeated-kicking performance decrement (fatigue index), kicking consistency, dynamic balance, inter-limb asymmetry, and kick accuracy were evaluated as additional performance outcomes.

#### 2.4.1 Dynamic balance

Dynamic balance was assessed using the Lower Quarter Y-Balance Test according to established procedures (10–12). Performance was expressed as the normalized composite score, calculated as the sum of the anterior, posteromedial, and posterolateral reach distances divided by three times the participant's limb length and multiplied by 100, in accordance with the standard Y-Balance Test scoring method.

#### 2.4.2 Stimulus-to-contact time

Stimulus-to-contact time was assessed using the PowerKube™ SMART striking pad (Strike Research Ltd., Norwich, UK) operating in Speed Mode. The system measures the elapsed time between presentation of an auditory–visual stimulus and successful target contact, thereby providing an objective measure of stimulus-to-contact performance during a sport-specific kicking task. This outcome should therefore be interpreted as reflecting the interval between stimulus presentation and target contact rather than isolated movement time. In addition to temporal outcomes, the system records impact-related variables, including impact power and kinetic energy. According to the manufacturer's technical documentation, the system uses controlled-compression technology and ultra-high-speed transducers for real-time impact analysis; however, the sampling frequency (Hz) has not been publicly disclosed by the manufacturer (24).

#### 2.4.3 Kick accuracy

Kick accuracy was assessed using a researcher-developed Muay Thai-specific kicking-accuracy device. The system consisted of a target-mounted vibration sensor connected to a computerized recording system that automatically detected successful target contacts during standardized kicking trials. Participants performed 30 consecutive roundhouse kicks towards a fixed target positioned at a standardized height and distance while maintaining a consistent kicking cadence. Kick accuracy was calculated as the percentage of successful target contacts relative to the total number of kicks performed. The instrument demonstrated acceptable test–retest reliability (intraclass correlation coefficient [ICC] = 0.89) and good content validity based on expert evaluation (item-objective congruence [IOC] = 0.92), as established during the original instrument-development study (25).

#### 2.4.4 Kicking consistency

Kicking consistency was quantified as the coefficient of variation (CV, %) of the stimulus-to-contact time recorded for each valid roundhouse kick during the standardized repeated-kicking protocol. Specifically, the CV was calculated as the standard deviation divided by the mean stimulus-to-contact time across all valid kicks completed within each testing session and multiplied by 100 (CV = SD/Mean × 100). Thus, the continuous variable used to calculate kicking consistency was stimulus-to-contact time (s) rather than binary success/failure outcomes or kick counts. Lower CV values indicated greater movement consistency, reflecting reduced trial-to-trial variability in stimulus-to-contact performance across repeated kicking attempts.

#### 2.4.5 Repeated-kicking performance decrement (fatigue index)

Repeated-kicking performance was assessed using a Muay Thai-specific repeated roundhouse-kick protocol adapted from the conceptual framework of the Frequency Speed of Kick Test (FSKT**)** described by da Silva Santos and Franchini (21). The protocol consisted of five 10 s bouts of maximal alternating roundhouse kicks separated by 10 s passive recovery intervals.

The primary variables recorded were the number of kicks completed during each 10 s bout, the total number of kicks completed across all five bouts, and a performance decrement index (fatigue index) calculated as:

Decrement(%)=[1−(Totalkicks÷(Bestset×5))]×100

The performance decrement index was used as an indicator of repeated-kicking capacity across successive high-intensity efforts and should not be interpreted as a direct physiological measure of fatigue. Lower decrement values indicated a greater ability to maintain kicking performance throughout the repeated-kicking protocol.

#### 2.4.6 Standardization of testing procedures

Participants were instructed to avoid strenuous exercise for 24–48 h before each assessment, maintain consistent dietary and hydration practices, avoid caffeine consumption for 6–12 h, use the same footwear (or remain barefoot, where appropriate) during both testing sessions, and attend testing at approximately the same time of day. These procedures were implemented to minimise potential sources of external variability between PRE and POST assessments.

To minimise fatigue-related influences, outcome assessments were organised into two standardized testing blocks separated by a scheduled recovery period. The first block consisted of the Lower Quarter Y-Balance Test, followed by the stimulus-to-contact time assessment. After the scheduled recovery period, participants completed the kick-accuracy assessment, followed immediately by the repeated-kicking protocol, from which repeated-kicking performance decrement (fatigue index) and kicking consistency were derived. The same testing sequence was applied to all participants during both PRE and POST assessments to ensure procedural consistency throughout the study.

### 2.5 Statistical analyses

All statistical analyses were performed using IBM SPSS Statistics (version 29.0; IBM Corp., Armonk, NY, USA) and R (version 4.3.3; R Foundation for Statistical Computing, Vienna, Austria). Linear mixed-effects models were fitted using restricted maximum likelihood (REML) estimation to examine the main effects of group and time, together with the group × time interaction, with participant included as a random intercept to account for repeated measurements and within-subject dependence (14). A compound-symmetry covariance structure was specified because only two assessment time points (PRE and POST) were obtained for each participant, providing an appropriate and parsimonious approach for modelling within-subject correlation.

Model assumptions were evaluated by visual inspection of residual distributions and assessment of homoscedasticity. Where necessary, outcome variables were transformed to improve model fit; however, untransformed descriptive statistics are presented to facilitate interpretation.

Results are reported as estimated marginal means, corresponding 95% confidence intervals (95% CIs), and standardized mean differences (Hedges' *g*) to facilitate interpretation of the magnitude and precision of the observed intervention effects. To account for multiplicity, the six prespecified group × time interaction tests corresponding to the six Muay Thai-specific performance outcomes (stimulus-to-contact time, kick accuracy, repeated-kicking performance decrement, kicking consistency, dynamic balance, and inter-limb asymmetry) were treated as a single family of tests. The Benjamini–Hochberg false discovery rate (FDR) procedure was applied across these six interaction tests (15). Both unadjusted and Benjamini–Hochberg-adjusted *p* values are reported in Table 2, and statistical significance was interpreted with reference to the adjusted *p* values. Statistical significance was set at *p* < 0.05 (two-tailed).

Statistical analyses were performed by the principal investigator in accordance with a prespecified statistical analysis plan using standardized analytical procedures. Although these procedures were applied consistently throughout the study, the absence of an independent blinded statistician is acknowledged as a potential source of analytical bias and should be considered when interpreting the findings.

No missing outcome data occurred because all allocated participants completed the intervention and all outcome assessments. Consequently, no data imputation procedures were required, and all analyses were performed using the complete dataset.

## 3 Results

### 3.1 Baseline characteristics

Following screening of 44 athletes, five were excluded before allocation. The remaining 39 eligible athletes were allocated to the experimental group (EXP, *n* = 20) or the control group (CON, *n* = 19). All allocated participants completed the six-week study period and post-intervention assessment; no participants withdrew after allocation, were lost to follow-up, discontinued their assigned training programme, or were excluded from the final statistical analyses.

Baseline characteristics of participants in the experimental and control groups are presented descriptively in Table 1 to provide contextual information before the intervention. Participant characteristics, including age, height, body mass, leg length, training experience, number of fights, and weekly training frequency, are summarised for each group. More detailed participant characteristics, including weight class, competition level, medal status, and dominant leg, are presented in Supplementary Table S1.

**Table 1:** Baseline characteristics of participants in the experimental (EXP) and control (CON) groups.

Variable	EXP (*n* = 20)	CON (*n* = 19)	*p*-value
Age (years)	19.2 ± 3.3	19.6 ± 3.0	0.710
Height (cm)	172.2 ± 4.6	172.3 ± 5.0	0.942
Body mass (kg)	58.0 ± 6.6	58.0 ± 5.4	0.980
Leg length (cm)	87.2 ± 7.3	86.8 ± 7.5	0.880
Training experience (years)	4.4 ± 1.6	5.1 ± 1.7	0.199
Number of fights (*n*)	26.6 ± 4.2	25.5 ± 4.9	0.491
Training frequency (sessions·week^−1^)	5.2 ± 1.0	5.2 ± 0.8	0.833

Values are presented as mean ± SD. Baseline between-group comparisons were performed for descriptive purposes only; no statistically significant differences were observed between groups for any variable (all *p* > 0.05).

### 3.2 Intervention adherence and safety

Intervention adherence was high in both groups. In the experimental group, 19 participants completed all 18 planned training sessions, whereas one participant missed a single session during week 4 because of a scheduled dental appointment. In the control group, one participant missed two scheduled training sessions during week 2 owing to mandatory participation in an educational camp organised by their educational institution. No participant exceeded the predefined non-adherence threshold (>15% missed sessions), and no participant was excluded from the final analyses because of non-adherence.

No serious intervention-related adverse events were observed. Mild, transient muscle soreness was occasionally reported in the experimental group during the early weeks of training but resolved spontaneously without medical treatment or interruption of the intervention. No participant withdrew from the study because of an adverse event.

### 3.3 Primary performance outcomes

**Figure 2:**
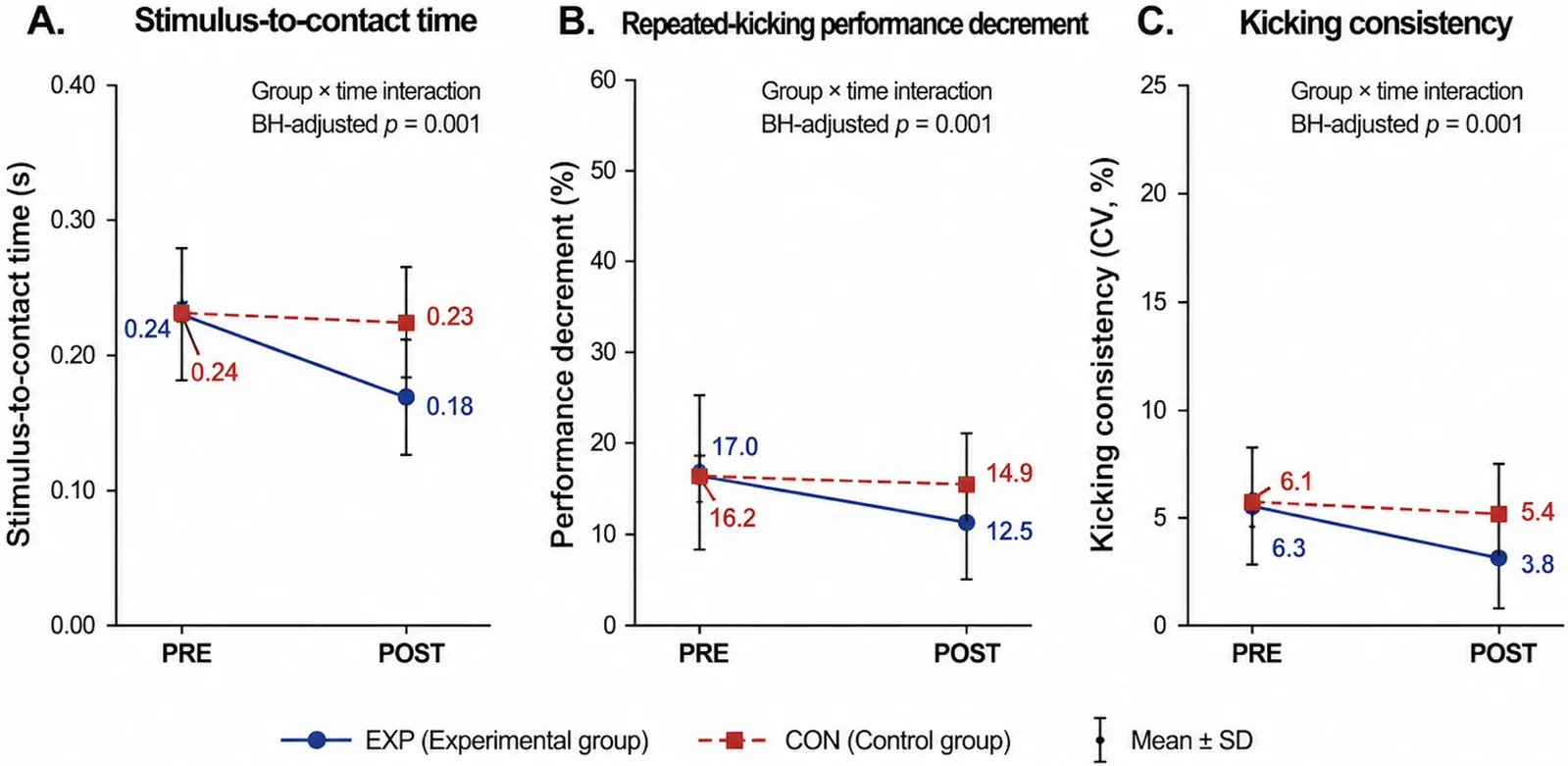
Changes in primary Muay Thai-specific performance outcomes from PRE to POST in the experimental (EXP) and control (CON) groups. **(A)** Stimulus-to-contact time; **(B)** repeated-kicking performance decrement; and **(C)** kicking consistency, expressed as the coefficient of variation (CV) of stimulus-to-contact time. Points and error bars represent mean ± SD. Group × time interaction *p* values were adjusted using the Benjamini–Hochberg false discovery rate procedure.

Changes in the primary Muay Thai-specific performance outcomes are summarised in Table 2 and illustrated in Figure 2. Significant group × time interactions were observed for all primary outcomes (all BH-adjusted *p* = 0.001), indicating greater improvements in the experimental group than in the control group.

**Table 2:** Changes in Muay Thai-specific kicking performance outcomes following the six-week intervention in the experimental (EXP) and control (CON) groups.

Variable	EXP PRE (mean ± SD)	EXP POST (mean ± SD)	CON PRE (mean ± SD)	CON POST (mean ± SD)	Δ difference (EXP − CON)	95% CI	Hedges' *g*	*P* (time)	*P* (group × time)	BH-adjusted *P* (group × time)
Stimulus-to-contact time (s)	0.24 ± 0.03	0.18 ± 0.04	0.24 ± 0.03	0.23 ± 0.04	−0.05	[−0.07, −0.03]	1.45	<0.001	<0.001	0.001
Kick accuracy (%)	82.9 ± 6.4	85.2 ± 6.8	82.3 ± 6.7	83.5 ± 6.9	+1.3	[0.2, 2.4]	0.28	0.031	0.048	0.072
Repeated-kicking performance decrement (%)	17.0 ± 5.3	12.5 ± 4.1	16.2 ± 5.5	14.9 ± 4.8	−3.2	[−4.4, −2.0]	0.95	<0.001	<0.001	0.001
Kicking consistency (CV, %)	6.3 ± 2.1	3.8 ± 2.6	6.1 ± 2.0	5.4 ± 2.3	−1.8	[−2.6, −1.0]	0.88	<0.001	<0.001	0.001
Dynamic balance (%)	92.0 ± 5.3	92.9 ± 5.6	91.6 ± 5.5	92.2 ± 5.8	+0.3	[−0.8, 1.4]	0.10	0.142	0.221	0.221
Inter-limb asymmetry (%)	7.2 ± 3.0	5.9 ± 2.6	6.8 ± 3.2	6.1 ± 3.0	−0.6	[−1.4, 0.2]	0.25	0.061	0.118	0.142

Values are presented as mean ± SD. Δ (EXP − CON) represents the between-group difference in change scores. Effect sizes are expressed as Hedges' *g*. The Benjamini–Hochberg false discovery rate (FDR) procedure was applied across the six prespecified group × time interaction tests. Statistical significance after multiplicity adjustment should be interpreted with reference to the BH adjusted *P* values. Lower values indicate better performance for stimulus-to-contact time, repeated-kicking performance decrement (fatigue index), and kicking consistency, expressed as the coefficient of variation (CV, %).

For stimulus-to-contact time, the EXP group demonstrated a substantial reduction from PRE to POST (0.24 ± 0.03 s to 0.18 ± 0.04 s), whereas the CON group showed only a modest reduction over the same period (0.24 ± 0.03 s to 0.23 ± 0.04 s). This resulted in a significant between-group difference in change scores (Δ = −0.05 s; 95% CI [−0.07, −0.03]; Hedges' *g* = 1.45).

For repeated-kicking performance decrement (fatigue index), used as an indicator of repeated-kicking capacity, the EXP group demonstrated a marked reduction from PRE to POST (17.0 ± 5.3% to 12.5 ± 4.1%), whereas the CON group showed a smaller reduction (16.2 ± 5.5% to 14.9 ± 4.8%). This resulted in a significant group × time interaction (Δ = −3.2%; 95% CI [−4.4, −2.0]; Hedges' *g* = 0.95).

Similarly, kicking consistency, expressed as the coefficient of variation (CV, %), improved substantially in the EXP group, with values decreasing from 6.3 ± 2.1% to 3.8 ± 2.6%, whereas the CON group changed from 6.1 ± 2.0% to 5.4 ± 2.3%. The between-group difference in change scores was statistically significant (Δ = −1.8 percentage points; 95% CI [−2.6, −1.0]; Hedges' *g* = 0.88).

To improve transparency regarding the repeated-kicking protocol, each participant completed five standardized 10 s bouts of maximal alternating roundhouse kicks during both the PRE and POST assessments. Per-bout and total kick counts are presented in Supplementary Table S2. Across the 39 participants and both assessment occasions, a total of 390 bouts comprising 5,367 valid kicks were completed and analysed. The numbers of valid kicks recorded in Bouts 1–5 were 1,089, 1,089, 1,060, 1,071, and 1,058, respectively, corresponding to mean ± SD values of 13.96 ± 1.14, 13.96 ± 1.25, 13.59 ± 1.09, 13.73 ± 1.22, and 13.56 ± 1.06 kicks per bout, respectively. Across all 390 bouts, the overall mean was 13.76 ± 1.16 kicks per bout. These counts characterize the repeated-kicking workload, while repeated-kicking performance decrement (fatigue index) and kicking consistency were calculated from the corresponding performance data as described in the Methods.

Figure 2 illustrates a consistent pattern of improvement across all primary outcomes in the experimental group, with clear separation between groups at POST testing.

### 3.4 Secondary performance outcomes

**Figure 3:**
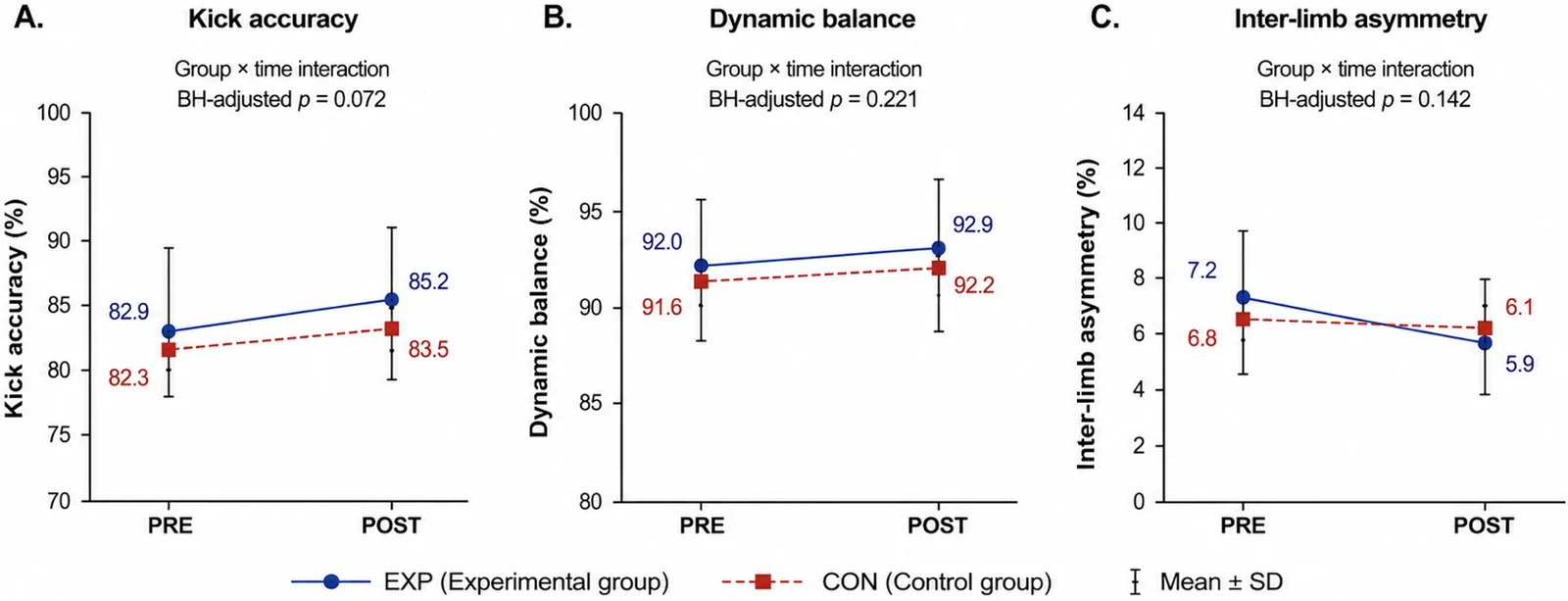
Changes in secondary Muay Thai-specific performance outcomes from PRE to POST in the experimental (EXP) and control (CON) groups. (A) Kick accuracy, (B) dynamic balance, and (C) inter-limb asymmetry. Points and error bars represent mean ± SD. The group × time interaction for kick accuracy did not remain statistically significant after Benjamini–Hochberg adjustment (BH-adjusted *p* = 0.072). No statistically significant group × time interactions were observed for dynamic balance (BH-adjusted *p* = 0.221) or inter-limb asymmetry (BH-adjusted *p* = 0.142).

Secondary performance outcomes are presented in Table 2 and Figure 3.

For kick accuracy, a small group × time interaction was observed (unadjusted *p* = 0.048; BH-adjusted *p* = 0.072), accompanied by a small standardized effect size (Hedges' *g* = 0.28). However, this interaction did not remain statistically significant after Benjamini–Hochberg adjustment and should therefore be interpreted with caution. The EXP group demonstrated a small increase in kick accuracy from 82.9 ± 6.4% at PRE to 85.2 ± 6.8% at POST, whereas the CON group showed a smaller increase from 82.3 ± 6.7% to 83.5 ± 6.9%.

No statistically significant group × time interactions were observed for dynamic balance (BH-adjusted *p* = 0.221) or inter-limb asymmetry (BH-adjusted *p* = 0.142). Both groups demonstrated minimal changes throughout the intervention period, with effect sizes remaining within the trivial-to-small range. As illustrated in Figure 3, trajectories for dynamic balance and inter-limb asymmetry remained largely parallel between groups, indicating that the intervention did not meaningfully influence these outcomes.

The overall distribution of categorical participant characteristics, including dominant leg, medal status, and competition level, is summarised in Supplementary Table S3. Collectively, these findings indicate that the observed training adaptations were primarily specific to repeated-kicking performance outcomes rather than broader measures of balance, limb symmetry, or kick accuracy after adjustment for multiple comparisons.

## 4 Discussion

### 4.1 Effects of the combined tire-squat and explosive-kicking programme on roundhouse-kick performance

This study examined the effects of a six-week combined tire-squat and explosive-kicking programme on multiple dimensions of roundhouse-kick performance in highly trained Muay Thai athletes. Overall, the findings largely supported the study hypothesis, with the experimental group demonstrating greater improvements in stimulus-to-contact time, repeated-kicking capacity, and kicking consistency than the control group, accompanied by moderate-to-large effect sizes (3, 7, 8). In contrast, the improvement in kick accuracy was modest and should be interpreted with caution because it did not remain statistically significant after adjustment for multiple comparisons. No meaningful changes were observed in dynamic balance or inter-limb asymmetry (10–12). Collectively, these findings suggest that the combined tire-squat and explosive-kicking programme was associated with favourable adaptations in selected sport-specific performance outcomes, particularly those related to rapid repeated kicking, while exerting limited influence on broader neuromuscular characteristics, including dynamic balance and inter-limb symmetry (18–23).

The substantial reduction in stimulus-to-contact time observed in the experimental group likely reflects improvements in movement execution speed, rate of force development, and neuromuscular coordination. Effective roundhouse-kick performance depends on rapid force transmission through the kinetic chain, requiring coordinated proximal-to-distal sequencing of the hip, knee, and ankle joints. Previous biomechanical studies have identified segmental coordination and explosive lower-limb force production as key determinants of kicking velocity and movement execution in combat sports (26, 27). However, a shorter stimulus-to-contact time should not be interpreted as evidence of greater impact force because direct biomechanical or instrumented impact-force measurements were not obtained in the present study. Accordingly, the observed improvement is more appropriately interpreted as an enhancement in movement execution speed rather than an increase in striking force. These findings are consistent with previous evidence demonstrating that resistance-based and neuromuscular training can improve sport-specific performance when the training stimulus closely reflects the mechanical and temporal characteristics of the target movement (27, 28). In the present study, combining progressive tire-squat exercise with explosive roundhouse-kick practice may have facilitated the transfer of lower-body strength adaptations to sport-specific kicking performance. This interpretation is consistent with the principle of dynamic correspondence, whereby training adaptations are optimized when the biomechanical and temporal characteristics of the exercise closely resemble those of the target skill (28–30). Nevertheless, because the intervention incorporated both progressive tire-squat exercise and additional explosive kicking practice, the independent contribution of each training component cannot be determined.

The present findings are also broadly consistent with previous investigations of elastic-resistance and other field-based neuromuscular training methods in combat sports, which have reported improvements in kicking speed, explosive performance, and movement-specific power development. Although elastic-resistance training and tire-squat exercise differ in their loading characteristics, both approaches aim to enhance lower-limb force production and neuromuscular coordination while maintaining a high degree of movement specificity (29–31). Furthermore, systematic reviews and meta-analyses have consistently demonstrated the effectiveness of resistance and neuromuscular training for improving speed, power, and sport-specific performance across athletic populations (29–31). The present findings extend this body of evidence by demonstrating that a practical combined training programme, incorporating progressive tire-squat exercise together with explosive roundhouse-kick practice, was associated with improvements in selected sport-specific performance outcomes in highly trained Muay Thai athletes.

The reduction in repeated-kicking performance decrement observed in the experimental group indicates an enhanced ability to maintain repeated-kicking performance across successive high-intensity bouts, a fundamental physical requirement in intermittent combat sports (1, 2). Accordingly, the repeated-kicking performance decrement index should be interpreted as an indicator of repeated-kicking capacity under standardized testing conditions rather than as a direct physiological measure of fatigue. Fatigue during repeated high-intensity exercise is multifactorial and may involve both central and peripheral mechanisms, including metabolic disturbances, impaired excitation–contraction coupling, and reduced motor-unit activation (32). However, because these mechanisms were not directly assessed in the present study, their contribution to the observed changes in repeated-kicking performance decrement cannot be determined.

The repeated-kicking protocol was adapted from the conceptual framework of the Frequency Speed Kick Test described by da Silva Santos and Franchini (21) and modified to better reflect the technical demands of repeated Muay Thai roundhouse kicks. Previous studies have demonstrated that high-intensity interval training can improve repeated-effort performance through adaptations in metabolic efficiency, buffering capacity, and neuromuscular function (33, 34). Similarly, the combined training programme, which incorporated progressive tire-squat exercise together with repeated explosive kicking under relatively short recovery intervals, may have promoted physiological and neuromuscular adaptations that enhanced participants' ability to maintain repeated-kicking performance throughout the standardized repeated-kicking protocol (33–35). The present findings are also consistent with previous evidence demonstrating that Muay Thai-specific high-intensity conditioning can improve sport-specific performance capacities in highly trained athletes, particularly those associated with repeated high-intensity kicking performance (36). Because the experimental intervention incorporated both progressive tire-squat exercise and repeated explosive kicking practice, however, the independent contribution of each training component cannot be determined. The observed adaptations should therefore be interpreted as reflecting the effects of the combined training programme rather than either component in isolation.

These adaptations may also contribute to maintaining movement quality during repeated high-intensity efforts by improving the efficiency and stability of motor-pattern organisation (36, 37). This interpretation is supported by the marked improvement in kicking consistency observed in the experimental group (37–39). Greater kicking consistency reflects enhanced temporal stability and movement control across repeated efforts rather than reliance on isolated peak performances (38–40). Previous investigations have suggested that neuromuscular training can reduce unnecessary movement variability by promoting more efficient coordination strategies (31, 38–40). In the present study, repeated explosive kicking practice incorporated within the combined training programme may have reinforced task-specific motor patterns, thereby contributing to improved movement consistency across successive kicking efforts (41). Although a modest improvement in kick accuracy was also observed, this finding should be interpreted cautiously because it was not consistently supported following adjustment for multiple comparisons, and its practical significance therefore remains uncertain.

### 4.2 Task specificity of the training adaptations

Although the repeated-kicking protocol was designed to reflect sport-specific movement patterns, it does not fully reproduce the complex physiological, tactical, and perceptual demands of an actual Muay Thai bout. Therefore, the fatigue index should be interpreted as a standardized indicator of repeated-kicking capacity under controlled testing conditions rather than as a direct measure of competitive performance. Accordingly, the observed improvements are best interpreted as reflecting enhanced repeated-kicking performance under standardized testing conditions. Future studies should determine whether these adaptations translate into improved performance during simulated or official Muay Thai competition, while also incorporating broader sport-specific performance indicators and technical skill assessments to better establish the ecological validity of field-based training interventions (42).

In contrast, dynamic balance and inter-limb asymmetry did not demonstrate meaningful improvements following the intervention. This finding is consistent with the specificity of the training stimulus, which primarily emphasized repeated explosive force production rather than postural control or bilateral symmetry (31, 38–40, 43, 44). Improvements in dynamic balance generally require more targeted proprioceptive or stability-oriented interventions, including perturbation-based and multidirectional balance exercises (38–40, 43, 44). Furthermore, participants in the present study were already highly trained and likely possessed relatively high baseline balance ability, thereby limiting the potential for further improvement because of ceiling effects (10–12, 38, 43). A similar explanation may apply to inter-limb asymmetry, as baseline performance was already relatively symmetrical, reducing the likelihood of detecting substantial changes over the six-week intervention period (21–23, 43, 44).

Taken together, these findings indicate that the observed adaptations were highly task-specific. From a practical perspective, the combined tire-squat and explosive-kicking programme may be useful for improving repeated-kicking performance but should be complemented with targeted balance, proprioceptive, or unilateral training when enhancing dynamic balance or reducing inter-limb asymmetry is a primary training objective.

### 4.3 Limitations

Several limitations of the present study should be acknowledged. First, although the six-week intervention was sufficient to induce short-term performance-related adaptations, it may not have been long enough to detect slower-developing changes in outcomes such as dynamic balance or inter-limb asymmetry. Second, no direct biomechanical assessments, including three-dimensional motion analysis, force-platform measurements, or instrumented impact-force testing, were performed. Consequently, the mechanisms underlying the observed performance improvements could not be fully characterized, and no definitive conclusions can be drawn regarding changes in impact force or technical execution.

Third, although the parallel-group controlled design strengthened the internal validity of the study, the sample comprised a relatively small feasibility-based sample of highly trained male Muay Thai athletes recruited under practical field conditions from a single competitive system. Accordingly, the findings should be generalized cautiously to female athletes, other age groups, recreational practitioners, and athletes from different striking disciplines.

Fourth, no formal *a priori* sample-size calculation was performed because reliable preliminary effect-size estimates for comparable Muay Thai-specific interventions were unavailable during the study-planning stage. Consequently, the study employed a feasibility-based sample that reflected the practical constraints of recruiting highly trained athletes for a controlled field-based intervention. Although this approach was appropriate for the present research context, the relatively modest sample size may have limited the precision of estimates for smaller treatment effects, particularly for kick accuracy. Accordingly, findings related to outcomes with small effect sizes should be interpreted cautiously and regarded as exploratory rather than confirmatory.

Fifth, several methodological limitations associated with participant allocation and outcome assessment should be considered. Participants were allocated using a manual lottery-based allocation procedure, and no formal allocation concealment mechanism was implemented. Consequently, the possibility of selection bias cannot be completely excluded, although predefined eligibility criteria, standardized participant screening, and a prespecified allocation procedure were applied consistently to minimise this risk. Furthermore, complete blinding was not feasible because of the nature of the intervention. Participants, training personnel, the outcome assessor, and the statistical analyst were aware of group allocation throughout the study. Outcome assessments and statistical analyses were performed by the principal investigator using standardized procedures and a prespecified statistical analysis plan; however, the absence of independent blinded outcome assessment and statistical analysis may have introduced measurement and analytical bias that cannot be entirely excluded. These potential sources of bias should therefore be considered when interpreting the present findings.

In addition, although session rating of perceived exertion (session RPE) was systematically monitored to guide progression of the experimental intervention, comparable internal training-load data were not collected in the control group because participants continued their usual training under their regular coaching programmes. Consequently, differences in overall internal training load between groups could not be quantified.

Furthermore, although the testing battery was organized into two standardized assessment blocks separated by a scheduled recovery period to minimize fatigue-related influences, the fixed assessment sequence may still have introduced residual order effects. Future studies should consider fully counterbalanced testing orders or separate assessment sessions to further reduce this potential source of bias.

Sixth, the study was not prospectively registered. Although it was designed, conducted, and reported in accordance with accepted methodological principles for controlled intervention studies, prospective trial registration was not completed before participant enrolment. This should therefore be acknowledged as a limitation with respect to reporting transparency.

Finally, although the intervention was designed to induce chronic adaptations through repeated training over six weeks, the inclusion of explosive roundhouse-kick practice within each intervention session means that a contribution from acute post-activation performance enhancement (PAPE) cannot be completely excluded. Consequently, the observed findings primarily reflect chronic training adaptations but may also include a small contribution from acute performance enhancement. Moreover, because the experimental intervention combined progressive tire-squat exercise with explosive roundhouse-kick practice, the independent contribution of each training component cannot be determined. Future studies should employ factorial or component-specific experimental designs, together with prospective *a priori* sample-size estimation and comprehensive biomechanical and physiological assessments, to distinguish the individual effects of each training component and further improve the precision of treatment-effect estimates.

### 4.4 Future directions

Future studies should investigate the longer-term effects of combined resistance- and explosive-kicking training on Muay Thai-specific performance. Such studies should incorporate comprehensive biomechanical, physiological, and neuromuscular assessments, including three-dimensional motion analysis, force-platform measures, instrumented impact-force testing, and relevant markers of neuromuscular function, to clarify the mechanisms underlying changes in stimulus-to-contact time, repeated-kicking decrement, and variability in kicking performance. Studies involving female athletes, athletes from different competitive standards, and participants from other striking disciplines would also help establish the generalizability of the present findings across broader athletic populations.

Future controlled trials should use prospectively registered protocols, pre-specified outcome hierarchies, *a priori* sample-size estimation, and transparent multiplicity-adjustment plans. Comprehensive monitoring of both internal and external training loads should be implemented across all study groups, while independent blinded outcome assessment and statistical analysis should be used whenever feasible. Importantly, future designs should match overall training exposure between groups and use factorial or component-specific comparisons to distinguish the independent and interactive effects of tire-squat exercise, explosive roundhouse-kick practice, and other training elements included in the intervention package.

Future investigations should also distinguish chronic training adaptations from acute post-activation performance enhancement (PAPE) responses. This could be achieved through standardized washout periods before testing, repeated assessment across the intervention period, and separate acute and longitudinal testing sessions. Such approaches would provide a clearer understanding of the time course and mechanisms through which combined resistance- and task-specific kicking training may influence Muay Thai performance.

Consistent with the emerging translational agenda for combat-sport research, future studies should combine rigorous controlled longitudinal designs with standardized intervention reporting, mechanistic assessment, athlete-safety monitoring, and collaboration among researchers, coaches, and sporting organizations. These approaches may strengthen the reproducibility, practical relevance, and safe translation of Muay Thai-specific training interventions into real-world competitive settings (45).

Finally, future studies should interpret intervention effects within a structured training-system perspective. Exercise selection, task-specific kicking practice, loading progression, coach supervision, technical feedback, safety procedures, athlete adherence, and implementation conditions may jointly influence observed adaptations. Accordingly, research designs should document these interacting components in sufficient detail and avoid attributing training effects to a single exercise component when the intervention is delivered as a combined programme (46).

### 4.5 Practical applications

The present findings indicate that the combined tire-squat and explosive-kicking programme was associated with improvements in selected measures of roundhouse-kick performance in highly trained Muay Thai athletes, particularly stimulus-to-contact time, repeated-kicking capacity (as reflected by a lower performance decrement index), and kicking consistency. These findings may assist coaches and practitioners in designing sport-specific conditioning programmes that incorporate progressive lower-limb resistance exercise together with explosive kicking practice under controlled training conditions.

The intervention consisted of three supervised training sessions per week over a six-week period using a progressive loading structure. During weeks 1–2, participants completed four sets of eight repetitions at moderate intensity (RPE 5–6). During weeks 3–4, the programme progressed to five sets of six repetitions at moderate-to-high intensity (RPE 6–7), followed by three explosive roundhouse kicks per leg performed within 20–30 s after each set. During weeks 5–6, participants completed six sets of five repetitions at high intensity (RPE 7–8), followed by a 5 s bout of maximal alternating roundhouse kicks, with 90–150 s of recovery between sets. External load was increased by approximately 5%–10% when participants completed all prescribed repetitions using appropriate technique and reported a session RPE of ≤7, whereas the load was reduced when movement quality deteriorated or perceived exertion became excessive.

Within the context of the present study, the combined programme was associated with improvements in stimulus-to-contact time, repeated-kicking capacity, and kicking consistency, whereas no meaningful changes were observed in dynamic balance or inter-limb asymmetry. In addition, the small improvement observed in kick accuracy should be interpreted with caution because it did not remain statistically significant after adjustment for multiple comparisons.

Because the intervention combined progressive tire-squat exercise with explosive roundhouse-kick practice, the independent contribution of each training component cannot be determined. Accordingly, the present findings should be interpreted as reflecting the effects of the combined training programme rather than those of either component in isolation. Further studies are warranted to determine whether these observed improvements translate into enhanced competitive performance or other sport-specific outcomes that were not evaluated in the present study.

## 5 Conclusions

In summary, the present study demonstrated that a six-week combined tire-squat and explosive-kicking programme was associated with improvements in stimulus-to-contact time, repeated-kicking capacity (as reflected by a lower repeated-kicking performance decrement index), and kicking consistency during repeated roundhouse-kick performance in highly trained Muay Thai athletes. These findings remained statistically significant after adjustment for multiple comparisons, indicating that the combined training programme was associated with improvements in selected sport-specific performance outcomes measured in the present study.

The observed adaptations were task-specific and should not be interpreted as evidence of generalized improvements across all aspects of athletic performance. No meaningful changes were observed in dynamic balance or inter-limb asymmetry, whereas the modest improvement in kick accuracy was not consistently supported following adjustment for multiple comparisons and should therefore be interpreted cautiously. Furthermore, because the experimental intervention combined progressive tire-squat exercise with explosive roundhouse-kick practice, the independent contribution of each training component cannot be determined. Accordingly, the present findings should be interpreted as reflecting the effects of the combined training programme rather than those of either intervention component in isolation.

These findings should also be interpreted in light of several study limitations, including the relatively small feasibility-based sample of highly trained male Muay Thai athletes, the absence of direct biomechanical and impact-force measurements, the lack of prospective trial registration, the short intervention period, and the inability to completely exclude acute post-activation performance enhancement (PAPE) effects.

Future studies employing factorial or component-specific experimental designs, together with comprehensive biomechanical, physiological, and neuromuscular assessments, are warranted to distinguish the individual contributions of tire-squat exercise and explosive kicking practice and to further clarify the mechanisms underlying the observed sport-specific performance adaptations. In addition, future investigations should determine whether the improvements observed under standardized testing conditions translate to competitive performance or other sport-specific outcomes that were not evaluated in the present study.

## Data Availability

The raw data supporting the conclusions of this article will be made available by the authors, without undue reservation.
