# Supplementary material for: Effects of a six-week combined tire-squat and explosive-kicking programme on roundhouse-kick performance in highly trained male Muay Thai athletes: a parallel-group controlled trial
Source: Front Sports Act Living. 2026 Aug 25;8:1885080. doi: 10.3389/fspor.2026.1885080 (PMC13550215; doi:10.3389/fspor.2026.1885080)
Supplement: Supplementary file 1 [file Table1.docx]

# Supplementary Table S1. Detailed participant characteristics

| **Participant ID** | **Group** | **Age (years)** | **Height (cm)** | **Body mass (kg)** | **Weight class** | **Leg length (cm)** | **Training experience (years)** | **Number of fights (n)** | **Training frequency (sessions·week⁻¹)** | **Dominant leg** | **Competition level** | **Medal** |
| --- | --- | --- | --- | --- | --- | --- | --- | --- | --- | --- | --- | --- |
| P01 | EXP | 23 | 165 | 51.71 | Super Flyweight | 82 | 6 | 22 | 4 | Left | Thailand National Games | Gold |
| P02 | CON | 23 | 166 | 52.62 | Bantamweight | 81 | 4 | 18 | 5 | Right | Thailand National Games | Silver |
| P03 | EXP | 25 | 170 | 60.85 | Lightweight | 83 | 3 | 36 | 6 | Left | Thailand National Games | Gold |
| P04 | CON | 24 | 168 | 58.73 | Super Featherweight | 80 | 9 | 32 | 6 | Right | Thailand National Games | Silver |
| P05 | EXP | 19 | 169 | 57.61 | Super Featherweight | 79 | 4 | 27 | 4 | Left | Thailand National Games | Silver |
| P06 | CON | 18 | 183 | 64.89 | Welterweight | 102 | 6 | 31 | 6 | Right | Thailand National Games | Silver |
| P07 | EXP | 17 | 176 | 63.42 | Super Lightweight | 95 | 5 | 27 | 6 | Right | Thailand National Youth Games | Gold |
| P08 | CON | 16 | 172 | 60.75 | Lightweight | 84 | 4 | 22 | 6 | Left | Thailand National Youth Games | Silver |
| P09 | EXP | 23 | 170 | 55.34 | Super Bantamweight | 82 | 5 | 30 | 6 | Right | Thailand National Games | Gold |
| P10 | CON | 21 | 170 | 56.97 | Featherweight | 81 | 4 | 19 | 4 | Right | Thailand National Games | Silver |
| P11 | EXP | 16 | 175 | 59.29 | Lightweight | 88 | 4 | 29 | 6 | Left | Thailand National Youth Games | Gold |
| P12 | CON | 16 | 172 | 59.76 | Lightweight | 85 | 7 | 30 | 5 | Right | Thailand National Youth Games | Silver |
| P13 | EXP | 25 | 171 | 56.83 | Featherweight | 87 | 9 | 31 | 6 | Right | Thailand National Games | Silver |
| P14 | CON | 24 | 174 | 59.42 | Lightweight | 86 | 8 | 33 | 4 | Left | Thailand National Games | Gold |
| P15 | EXP | 15 | 170 | 50.63 | Flyweight | 80 | 3 | 29 | 6 | Right | Thailand National Youth Games | Silver |
| P16 | CON | 17 | 170 | 54.79 | Super Bantamweight | 83 | 4 | 32 | 6 | Left | Thailand National Youth Games | Gold |
| P17 | EXP | 22 | 176 | 71.78 | Middleweight | 90 | 5 | 28 | 4 | Left | Thailand National Games | Gold |
| P18 | CON | 23 | 174 | 70.39 | Middleweight | 87 | 5 | 24 | 5 | Right | Thailand National Games | Gold |
| P19 | EXP | 20 | 168 | 52.62 | Bantamweight | 86 | 4 | 27 | 6 | Right | Thailand National Games | Silver |
| P20 | CON | 21 | 167 | 53.36 | Bantamweight | 84 | 5 | 22 | 4 | Left | Thailand National Games | Silver |
| P21 | EXP | 18 | 176 | 58.66 | Super Featherweight | 88 | 4 | 23 | 6 | Right | Thailand National Games | Gold |
| P22 | CON | 19 | 175 | 57.78 | Super Featherweight | 90 | 5 | 25 | 5 | Right | Thailand National Games | Silver |
| P23 | EXP | 18 | 178 | 58.19 | Super Featherweight | 101 | 3 | 26 | 4 | Left | Thailand National Games | Gold |
| P24 | CON | 19 | 168 | 56.25 | Featherweight | 81 | 4 | 21 | 6 | Right | Thailand National Games | Gold |
| P25 | EXP | 18 | 178 | 57.36 | Super Featherweight | 100 | 4 | 22 | 5 | Left | Thailand National Games | Gold |
| P26 | CON | 15 | 170 | 50.49 | Flyweight | 83 | 3 | 20 | 6 | Right | Thailand National Youth Games | Gold |
| P27 | EXP | 17 | 173 | 71.55 | Middleweight | 85 | 3 | 22 | 4 | Right | Thailand National Youth Games | Silver |
| P28 | CON | 17 | 176 | 60.81 | Lightweight | 99 | 4 | 25 | 5 | Left | Thailand National Youth Games | Gold |
| P29 | EXP | 16 | 175 | 59.86 | Lightweight | 84 | 3 | 21 | 6 | Right | Thailand National Youth Games | Silver |
| P30 | CON | 18 | 176 | 58.79 | Super Featherweight | 86 | 4 | 26 | 4 | Right | Thailand National Games | Gold |
| P31 | EXP | 18 | 170 | 52.13 | Super Flyweight | 84 | 4 | 27 | 6 | Left | Thailand National Games | Silver |
| P32 | CON | 24 | 170 | 56.72 | Featherweight | 85 | 8 | 33 | 6 | Right | Thailand National Games | Gold |
| P33 | EXP | 16 | 172 | 60.44 | Lightweight | 87 | 4 | 25 | 4 | Right | Thailand National Youth Games | Gold |
| P34 | CON | 17 | 165 | 45.81 | Mini Flyweight | 80 | 5 | 23 | 5 | Left | Thailand National Youth Games | Gold |
| P35 | EXP | 16 | 165 | 47.62 | Mini Flyweight | 78 | 3 | 21 | 6 | Right | Thailand National Youth Games | Silver |
| P36 | CON | 18 | 183 | 63.96 | Welterweight | 106 | 3 | 26 | 5 | Left | Thailand National Games | Silver |
| P37 | EXP | 18 | 165 | 48.93 | Light Flyweight | 81 | 4 | 24 | 4 | Left | Thailand National Games | Gold |
| P38 | CON | 22 | 174 | 60.14 | Lightweight | 86 | 5 | 23 | 6 | Right | Thailand National Games | Silver |
| P39 | EXP | 24 | 181 | 64.68 | Welterweight | 103 | 8 | 34 | 4 | Right | Thailand National Games | Gold |

*Note.* Individual participant characteristics are presented to provide detailed descriptive information and to support transparency and reproducibility of the study sample. Categorical variables include weight class, competition level, medal status, and dominant leg.
