# Supplementary material for: Effects of a six-week combined tire-squat and explosive-kicking programme on roundhouse-kick performance in highly trained male Muay Thai athletes: a parallel-group controlled trial
Source: Front Sports Act Living. 2026 Aug 25;8:1885080. doi: 10.3389/fspor.2026.1885080 (PMC13550215; doi:10.3389/fspor.2026.1885080)
Supplement: Supplementary file 2 [file Table2.docx]

Supplementary Table S2. Per-bout and total kick counts during the standardized repeated-kicking protocol at PRE and POST assessments (n = 39)

| **Assessment** | **Bout 1** | **Bout 2** | **Bout 3** | **Bout 4** | **Bout 5** | **Total kicks** | **Total kicks across all participants** |
| --- | --- | --- | --- | --- | --- | --- | --- |
| PRE | 13.79 ± 1.24 | 13.79 ± 1.30 | 13.44 ± 1.12 | 13.64 ± 1.25 | 13.23 ± 0.93 | 67.90 ± 2.53 | 2648 |
| POST | 14.13 ± 1.03 | 14.13 ± 1.20 | 13.74 ± 1.04 | 13.82 ± 1.21 | 13.90 ± 1.10 | 69.72 ± 2.41 | 2719 |

*Note.* Values for individual bouts and total kicks are presented as mean ± SD. Each participant completed five standardized 10-s bouts of maximal alternating roundhouse kicks, separated by 10-s passive recovery intervals, at both PRE and POST assessments. Total kicks represent the sum of Bouts 1–5 for each participant. Across both assessment occasions, 390 bouts comprising 5,367 valid kicks were analysed, corresponding to an overall mean of 13.76 kicks per bout.
