# Supplementary material for: Effects of a six-week combined tire-squat and explosive-kicking programme on roundhouse-kick performance in highly trained male Muay Thai athletes: a parallel-group controlled trial
Source: Front Sports Act Living. 2026 Aug 25;8:1885080. doi: 10.3389/fspor.2026.1885080 (PMC13550215; doi:10.3389/fspor.2026.1885080)
Supplement: Supplementary file 3 [file Table3.docx]

Supplementary Table S3. Distribution of categorical variables

| **Variable** | **Category** | **n (%)** |
| --- | --- | --- |
| Dominant leg | Left | 18 (46.2%) |
|  | Right | 21 (53.8%) |
| Medal | Gold | 22 (56.4%) |
|  | Silver | 17 (43.6%) |
| Competition level | National Games | 27 (69.2%) |
|  | Youth Games | 12 (30.8%) |

*Note*. Values are presented as n (%). Percentages are calculated using the total sample (n = 39) as the denominator.
